# Rapid Fabrication of Tungsten Oxide-Based Electrochromic Devices through Femtosecond Laser Processing

**DOI:** 10.3390/mi15060785

**Published:** 2024-06-14

**Authors:** Liqun Wang, Zihao Zhai, Longnan Li

**Affiliations:** 1 GPL Photonics Laboratory, State Key Laboratory of Luminescence Science and Technology, Changchun Institute of Optics, Fine Mechanics and Physics, Chinese Academy of Sciences, Changchun 130033, China; 2 Center of Materials Science and Optoelectronics Engineering, University of Chinese Academy of Sciences, Beijing 100049, China

**Keywords:** tungsten trioxide, electrochromism, femtosecond laser, rapid fabrication

## Abstract

The sol-gel method is a widely adopted technique for the preparation of tungsten trioxide (WO_3_) materials, favored for its cost-effectiveness and straightforward production procedures. However, this method encounters challenges such as prolonged annealing periods and limited flexibility in fabricating patterned WO_3_ films. This study introduces a novel approach that integrates femtosecond laser processing with the sol-gel method to enhance the fabrication of WO_3_ films. By adjusting polyvinylpyrrolidone (PVP) concentrations during sol-gel synthesis, precise control over film thickness and optimized film properties were achieved. The innovative technique significantly reduced the annealing time required to achieve an 80% transmittance rate from 90 min to 40 min, marking a 56% decrease. Laser processing increased the surface roughness of the films from Sa = 0.032 to Sa = 0.119, facilitating enhanced volatilization of organics during heat treatment. Additionally, this method improved the transmittance modulation of the films by 22% at 550 nm compared to unprocessed counterparts. This approach not only simplifies the manufacturing process but also enhances the optical efficiency of electrochromic devices, potentially leading to broader applications and more effective energy conservation strategies.

## 1. Introduction

Electrochromic (EC) devices can reversibly alter their optical properties, such as absorbance, transmittance, and reflectance, in response to applied voltage [1]. These devices have critical applications in various fields, including large-scale smart windows for buildings [2,3,4], automobiles [5], displays [6,7], electrochromic e-skins [8], and energy storage devices [9,10,11]. Among their capabilities, EC-based smart windows are particularly effective at blocking solar radiation, thus reducing indoor temperatures and promoting energy efficiency. Tungsten trioxide (WO_3_), a prominent cathode material in ECs, is distinguished by its superior visible light modulation and memory characteristics. It was one of the first EC materials to find practical application due to these properties [12,13]. The operation of WO_3_ involves the insertion or removal of positive ions and electrons under an external electric field, resulting in the redox conversion between W^6+^ and W^5+^ states, which in turn results in the coloring and bleaching of the film [14,15,16,17,18,19].

The fabrication of WO_3_ films for EC applications is achieved through various techniques including magnetron sputtering [20,21,22,23,24], thermal and electron beam evaporation [25,26,27,28,29], chemical vapor deposition (CVD) [30,31], sol-gel processes [32,33,34,35], and hydrothermal reactions [36,37,38,39,40,41,42]. Each method presents its own set of challenges regarding production costs, experimental conditions, and scalability. Magnetron sputtering, thermal and electron beam evaporation, and CVD methods, despite producing dense and uniform films, are limited by high production costs and demanding environmental conditions. Conversely, the hydrothermal method, while cost-effective, requires operation under high temperatures and pressures, making it complex and unsuitable for large-scale production. The sol-gel process has emerged as the industry standard due to its simplicity and cost-effectiveness [43,44,45,46,47,48]. Despite its advantages, the sol-gel process involves challenges such as the need for spin-coating precursor solutions onto conductive glass substrates followed by annealing at high temperatures [49], resulting in lengthy fabrication times. Additionally, existing WO_3_ fabrication methods typically produce either particles or continuous films, lacking the flexibility to pattern electrochromic devices selectively with WO_3_ layers, a limitation shared across all current fabrication techniques.

In recent years, lasers have garnered attention as a novel processing method due to their unique advantages. This technique, unlike traditional methods, does not require contact with the workpiece, thereby reducing the risk of contamination and mechanical damage [50]. Lasers enable precise localization, allowing for intricate patterns and modifications to be made with high accuracy. For instance, Cho et al. utilized a continuous-wave laser to selectively sinter WO_3_ nanoparticles [51]. This process was used for both patterning and annealing of WO_3_, demonstrating the capability of lasers to precisely control material properties at the nanoscale. By annealing WO_3_ on ITO glass substrates, the researchers were able to induce a transformation in the WO_3_ crystal state. This transformation is critical as it directly influences the electrochromic properties of the material. However, the study also highlighted several challenges. Poor adhesion observed can lead to delamination or failure of the device under mechanical stress. This presents a barrier to the durability and longevity of the electrochromic devices. Additionally, the transmittance modulation achieved through this method—defined as the difference in transmittance between the bleached state and the colored state—was relatively small.

In response to these challenges, this study proposes a novel approach combining femtosecond laser processing with the sol-gel method to enable flexible and precise patterning of WO_3_ precursor films. This technique not only allows for clear coloration and complete bleaching but also significantly reduces the annealing time by 56% (from 90 min to 40 min), without compromising the optical property. Moreover, the femtosecond laser-processed samples demonstrate a 22% higher transmittance modulation at 550 nm compared to unprocessed samples, addressing the limitations of the sol-gel method and potentially revolutionizing the fabrication process for electrochromic devices. This innovative method promises enhanced efficiency and responsiveness in electrochromic materials, paving the way for broader applications and more effective energy conservation strategies.

## 2. Materials and Methods

### 2.1. Materials and Chemicals

Tungstic acid (H_2_WO_4_, Aladdin, Shanghai, China), hydrogen peroxide solution (H_2_O_2_, XINBOTE, Tianjin, China, 30 wt% in H_2_O), polyvinylpyrrolidone ((C_6_H_9_NO)_n_, Aladdin, Shanghai, China) and sodium sulphate (Na_2_SO_4_, Aladdin, Shanghai, China) were used without further purification. Commercial Indium-Tin Oxide (ITO) coated glass (Southern China Xiang’s Science & Technology, Shenzhen, China, ITO glass-100 × 100 × 1.1 mm, 6 Ω/sq) was cut into appropriate sizes. The surface floating dust was washed off with deionized water, and the surface organic matter was removed by cleaning with acetone, ethanol, and water in a sonication bath (KQ-400DE, Kunshan Ultrasonic Instruments, Kunshan, China) for 10 min each, then dried with nitrogen (N_2_) flow.

### 2.2. Preparation of WO_3_ Precursor Film

**Figure 1:**
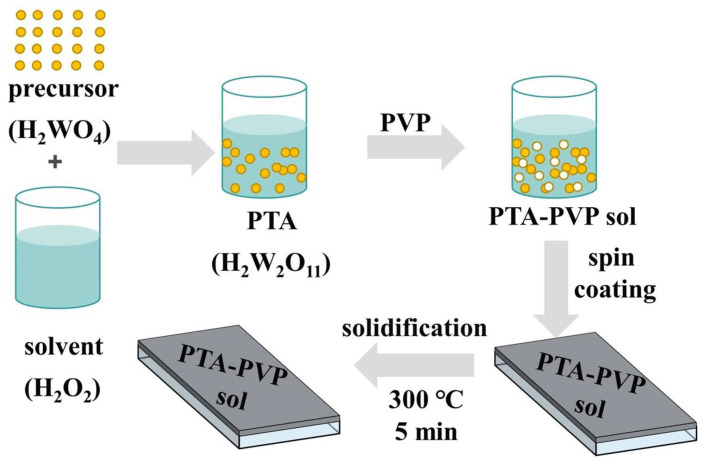
Preparation process of precursor film on ITO substrate by sol-gel method.

The precursor sol of WO_3_ was obtained using the peroxotungstic acid method (Figure 1). Tungstic acid powder and 30% hydrogen peroxide served as the reaction reagents, with ITO-conductive glass as the substrate. A magnetic stirrer (MS7-H550-Pro, DLAB Scientific Co., Ltd., Beijing, China) continuously agitated a mixture of 10 mL hydrogen peroxide (H_2_O_2_) and 1.5 g tungstic acid powder (H_2_WO_4_) at room temperature for five to seven days. This produced a translucent and clear PTA (peroxotungstic acid solution, chemical formula H_2_W_2_O_11_) solution. The PTA solution was then mixed with polyvinylpyrrolidone (PVP) at ratios of 0.5 g, 0.6 g, 0.7 g, and 0.8 g per 10 mL of PTA solution, respectively, and stirred for 1–2 h to obtain a transparent and uniform PTA-PVP gel. The addition of PVP increases the viscosity of the solution, which improves the quality of the film prepared by spin coating. A uniform WO_3_ precursor film was formed on the surface of ITO glass using a spin coating machine (KW-4A, SETCAS LLC, Beijing, China) at 2000 rpm for 20 s. Then, a thermal solidification process at 300 °C for 5 min was applied to the WO_3_ precursor film to enhance adhesion between the film and the substrate. Despite the longer preparation time required for the sol-gel method compared to magnetron sputtering and thermal evaporation processes, the exceptional stability of the PTA-PVP precursor solution [52,53] allows for mass production and storage of the precursor solution prior to the fabrication process. This enables the fabrication of WO_3_-based EC devices through spin-coating without the need for a vacuum environment, making it more time-efficient overall.

### 2.3. Femtosecond Laser Processing of WO_3_ Precursor Film

**Figure 2:**
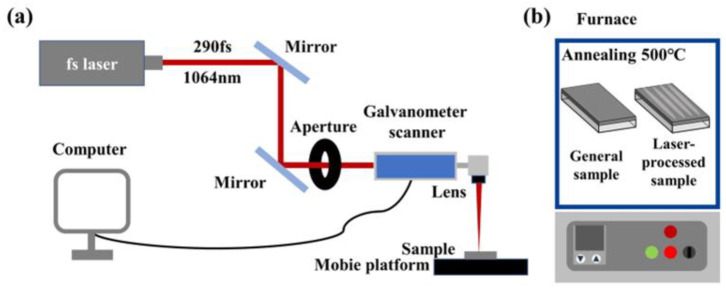
Schematic illustrations of (**a**) femtosecond laser system and (**b**) sample annealing method for WO_3_ film processing.

After the precursor film formation, the solidified sample was positioned on a one-dimensional moving platform, adjustable along the optical axis, for laser processing. A femtosecond laser system (PA-20 W, LIGHT CONVERSION, Vilnius, Lithuania), equipped with a galvanometer scanner and a computer-aided patterning system, was utilized for this purpose. The laser operated at a wavelength of 1064 nm, with a pulse duration of less than 290 femtoseconds. It had a variable repetition frequency and pulse energy, with a single pulse energy range of 3–400 μJ and a repetition rate range of 100 Hz to 1 MHz. The maximum power output was 20 W. To ensure precise laser processing, a steering mirror directed the laser beam, and an aperture filtered out any stray light. A focusing lens with an effective focal length of 165 mm concentrated the laser energy onto the sample surface, achieving a focal spot diameter of about 40 μm. The schematic diagram of the femtosecond laser processing setup is shown in Figure 2. Following laser processing, the sample underwent heat treatment at 500 °C in a muffle furnace (KSL-1200X-J, H-F Kejing Material Technology Co., Ltd., Hefei, China) to remove organic matter from the precursor film and improve the sample’s transmittance. For comparison, another solidified precursor film sample (referred to as the general sample) was directly annealed in a furnace at 500 °C for 2 h.

### 2.4. Characterization

The X-ray diffraction (XRD) test employed BRUKER’s D8 Focus equipment (D8 FOCUS, Bruker, MA, USA) operating at 40 kV and 40 mA. The scanning angle range was 20–80° with a step size of 0.02°/s. The surface morphology of the WO_3_ film was analyzed using the AURIGA-4506 scanning electron microscope (SEM) from Zeiss, Germany. The Raman spectrometer used was the LabRAM HR Evolution (Lab RAM HR Evolution, HORIBA FRANCE SAS, Longjumeau, France). The laser source utilized in Raman spectrometer was a 532 nm green light source. The spectrophotometer used was the Agilent Cary 5000 UV-Vis-NIR spectrophotometer (Cary 5000, US Agilent Technologies Co., Ltd., Santa Clara, CA, USA), covering a measurement range of 175–3300 nm, and equipped with Cary WinUV Version 6.2 Software for instrument control and data acquisition.

### 2.5. Electrochemical Measurement

A two-electrode system and a DC power supply (Keithley 2400, Keithley, Cleveland, OH, USA) were used for performance testing of the fabricated electrochromic devices at room temperature. The electrolyte solution employed was 0.3 mol/L sodium sulfate (Na_2_SO_4_), with the working electrodes being a WO_3_ film and a platinum wire (Pt) electrode, respectively. The coloring and bleaching voltages of WO_3_ were set for −3 V and +3 V, respectively.

## 3. Results and Discussions

### 3.1. Characterization of WO_3_ Precursor Film

The thickness of single-layer WO_3_ precursor film was precisely controlled by varying the concentration of PVP during the sol-gel synthesis. Experimental observations indicate a direct correlation between PVP concentration and film thickness. For instance, a PVP addition of 0.5 g per 10 mL of polyvinyl alcohol (PVA) solution results in a film thickness of approximately 586 nm. Increasing the PVP concentration to 0.8 g per 10 mL enhances the thickness to about 965 nm (Figure S1). At higher concentrations, the spin-coated film exhibits a non-uniform distribution on the substrate due to increased viscosity from higher PVP content. A low-viscosity sol under identical spin coating conditions reduces adhesion between the film and substrate, increasing the likelihood of hollowness. Conversely, high viscosity reduces ductility, hindering film formation.

**Figure 3:**
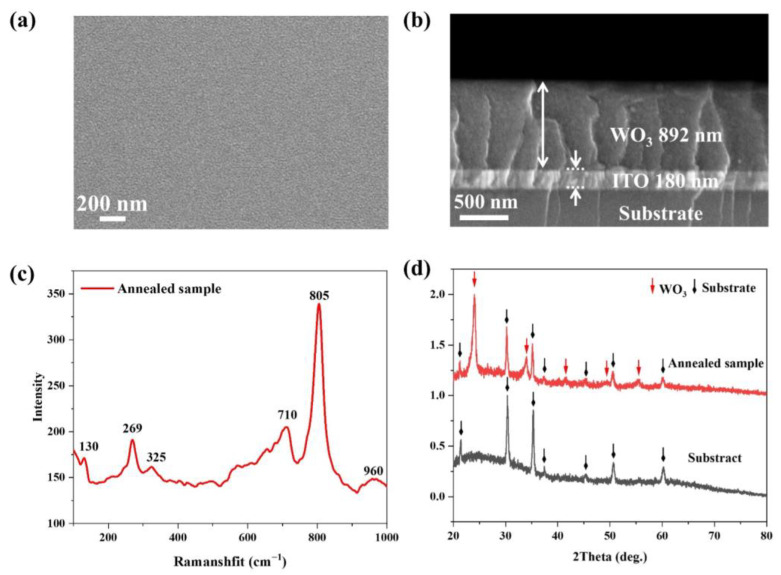
(**a**) Surface morphology of spin coated WO_3_ film; (**b**) Cross-section SEM image of annealed WO_3_ film; (**c**) Raman and (**d**) XRD spectra of the annealed sample (annealing at 500 °C for 2 h).

To optimize film properties, a PVP concentration of 0.7 g per 10 mL was chosen. The surface and cross-sectional morphologies of the resultant solidified PTA-PVP sol film were analyzed using SEM. As depicted in Figure 3a, the film surface is characterized by smoothness, density, and uniformity. The corresponding cross-sectional SEM image (Figure 3b) reveals a consistent film thickness of 892 nm, with a complete absence of pores or structural discontinuities, underscoring the high quality of the precursor film.

Figure 3c shows Raman spectroscopy results for the annealed general sample with WO_3_ film. The spectra exhibited characteristic peaks at 269, 325, 710, and 805 cm^−1^. The peaks at 269 and 325 cm^−1^ correspond to the bending vibrations of the W-O-W bond bridging oxygen, while the latter two are associated with tensile vibrations [54]. Additionally, the characteristic peak at 130 cm^−1^ indicates the formation of the O-O bond, and the peak at 960 cm^−1^ corresponds to the stretching mode of the W=O bond [55]. Further analysis of the phase composition of the prepared films was conducted using XRD. The XRD results, compared between the annealed WO_3_ film and bare ITO substrate samples, are presented in Figure 3d. The diffraction profiles of the thin film samples display sharp peaks at 2θ ≈ 24° and 34°, which correspond to <200> and <220> crystal planes of WO_3_ [56,57]. In addition, weaker diffraction characteristic peaks of WO_3_ appeared at 2θ ≈ 41.5°, 49.3°, and 55.4°, respectively. These peaks confirm the crystallization of the WO_3_ films after high-temperature annealing, substantiating their structural integrity and compositional homogeneity [58].

### 3.2. Femtosecond Laser Processing of PTA-PVP Sol Film

**Figure 4:**
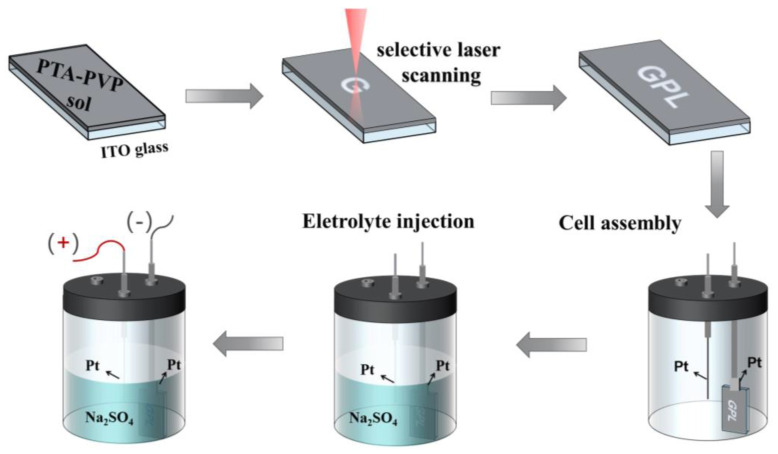
Schematic of selective laser annealing with femtosecond laser processing and electrochromic characterization process for WO_3_ precursor film.

We employ a femtosecond laser to selectively process solidified PTA-PVP sol films through photothermal chemical effects. Unlike the uniform heating of the muffle furnace (for the general sample), selective laser processing enables precise localized annealing of selected areas via a laser beam, which is critical for the selective fabrication of WO_3_ films. The selective laser processing and EC characterization of fabricated device include following steps: (1) Spin-coating of PTA-PVP sol-gel onto an ITO conductive glass substrate; (2) Laser processing of the precursor film to achieve flexible preparation of WO_3_; (3) Assembly of the electrochemical cell using platinum wire, platinum electrode, electrolytic cell, and sample to be tested; (4) Injection of a 0.3 mol/L Na_2_SO_4_ solution as the electrolyte solution; and (5) Application of electrical voltage to test the electrochromic (EC) performance, as depicted in Figure 4.

**Figure 5:**
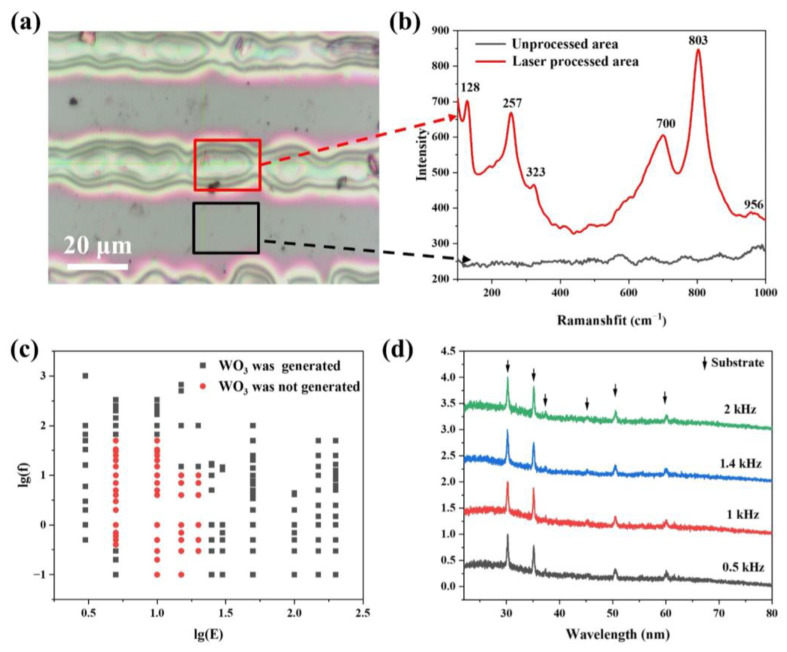
(**a**) Optical image and (**b**) Raman spectra of fs laser processed and unprocessed regions on solidified (heat treatment at 300 °C for 5 min) WO_3_ film. (**c**) Observation of WO_3_ as a function of laser frequency (*f*) and single pulse energy (*E*). The solidified precursor film processed with red dotted laser parameters generated WO_3_. (**d**) XRD spectra of samples processed at different laser frequencies.

To characterize laser-processed structures on the sample surface, Raman tests were conducted on the laser-processed and unprocessed regions of solidified precursor WO_3_ film (Figure S2) using the micro-area measurement function (Figure 5a). Optical microscopy images show the precursor film’s color transition from brown in the unprocessed area to green in the processed area, indicative of material conversion, where H_2_W_2_O_11_ decomposes into WO_3_ and H_2_O under the influence of laser heating, as represented by the following reaction [59,60]:

$$H_{2}W_{2}O_{11}\to {WO}_{3}{\left(H_{2}O\right)}_{n}\to {WO}_{3}+nH_{2}O$$

Raman spectroscopy revealed distinctive differences between the processed and unprocessed areas, as shown in Figure 5b. Consistent with the Raman spectrum after heat treatment, there are obvious WO_3_ characteristic peaks in the laser-processed region, which are located at 128, 257, 323, 700, 803, and 956 cm^−1^, respectively. Conversely, these Raman peaks were absent in the untreated precursor film, confirming the selective formation of WO_3_ in the laser-processed regions.

To ascertain the optimal fs-laser processing conditions for WO_3_ thin films, comprehensive parameter studies were conducted. Optimal conditions, identified by red dots in Figure 5c, were determined where the single pulse energy (*E*) ranged from 5 to 20 µJ (corresponding to the horizontal axis 0.7 to 1.3) and the repetition frequency (*f*) from 0.1 to 66.6 kHz (Corresponding to the longitudinal axis −1 to 1.82). By appropriately adjusting the laser scanning speed within these parameters, the precursor in the treated area was effectively converted into WO_3_. The laser processing parameters selected for subsequent analyses included a single pulse energy of 5 μJ, a repetition frequency of 1 kHz, a scanning speed of 5 mm/s, and a scanning line spacing of 0.04 mm.

Further phase composition analysis of the WO_3_ films, processed at varying laser frequencies (0.5 kHz, 1 kHz, 1.4 kHz, and 2 kHz), under conditions including a scanning line spacing of 0.04 mm, a scanning speed of 5 mm/s, and a single pulse energy of 5 μJ, showed no detectable crystalline peaks in XRD patterns (Figure 5d). This absence indicated the formation of amorphous WO_3_, due to the extremely rapid cooling rates afforded by the fs laser pulses, which prevent crystallization of the WO_3_ film [61,62]. To achieve a more stable crystallized WO_3_ film, the laser-processed film needs further annealing [63,64,65].

Subsequently, to investigate the EC performance of laser-processed solidified WO_3_ film, we patterned “GPL” and “H” characters on the surfaces. The WO_3_ coloring process involves the injection of cations and electrons into the film from both sides, leading to the reduction in W atoms and the appearance of a blue color in the film. Conversely, the bleaching process involves the extraction of cations and electrons from the film, oxidizing the W atoms and restoring the film to a transparent state. When Na_2_SO_4_ is used as the electrolyte, the specific discoloration mechanism is as follows [66]:

(1) $${WO}_{3}+x{Na}^{+}+xe^{-}↔{Na}_{x}{WO}_{3}$$

Furthermore, a crucial step in the EC process is ion insertion/extraction, which can be broken down into five stages: ion transfer from the electrolyte to the film surface; ion entry into the film and movement throughout it; ion embedding into the WO_3_ lattice; ion extraction from the WO_3_ lattice; and ion release from the membrane. As shown in Figure S3, when a negative voltage is applied, the laser-processed area is colored, and the “H” and “GPL” patterns appear on the film surface. Note that the laser processing was conducted immediately after the solidification process but before the annealing process. This sequence ensured that the unprocessed area remained non-transparent due to the carbonization of organic matter, including PVP, within the precursor film. Furthermore, the sample depicted in Figure S3 has not undergone the complete annealing process (Figure 2b). Conversely, when a positive voltage is applied, the laser-processed area is bleached, and the pattern disappears. The contrast between the colored state and the bleached state is readily apparent, and the material can be prepared in various patterns, rendering it suitable for the transmission of daily information and offering significant potential utility in fields such as displays.

### 3.3. Characterization of Laser Processed WO_3_ Film

**Figure 6:**
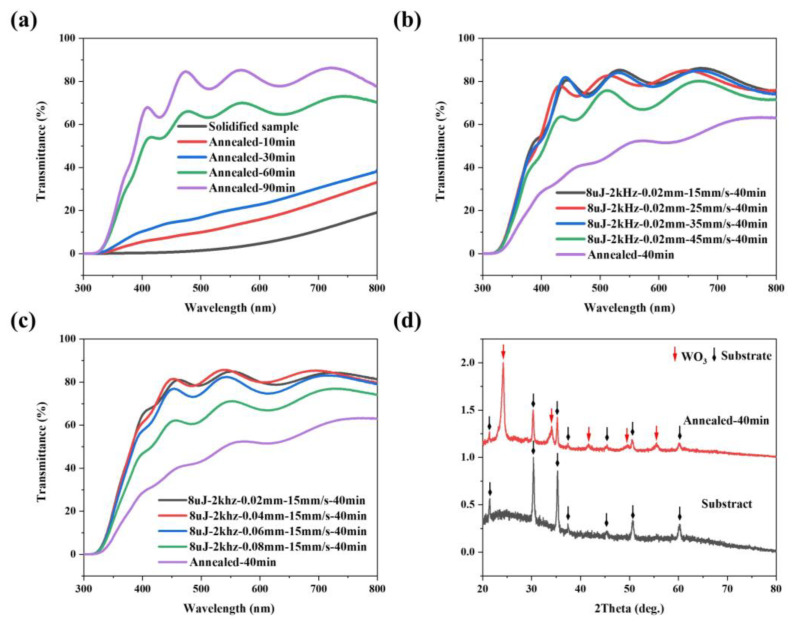
(**a**) Transmittance spectra of the unprocessed WO_3_ films as a function of annealing time at 500 °C; (**b**,**c**) Effect of laser processing parameters on transmittance of WO_3_ films with annealing time of 40 min at 500 °C; (**d**) XRD spectra of laser-processed sample (8 μJ-2 kHz-0.02 mm-15 mm/s) annealed at 500 °C for 40 min.

Laser processing alone cannot achieve WO_3_ films with high transmittance due to the inability of organic matter to be completely volatilized when the energy density of the film surface is low, and the film is susceptible to destruction when the energy density of the film surface is high. Conventionally, the removal of organic matter from the precursor film and the formation of crystalline WO_3_ is achieved through high-temperature annealing as shown in Figure 3d [67,68,69]. Figure 6a shows the transmittance spectra of general solidified WO_3_ film samples that underwent annealing at 500 °C for varying duration. As annealing time increased, organic matter gradually volatilized from the film, enhancing its transmittance. For the samples that have undergone the solidification process, the transmittance at λ = 550 nm is nearly zero. When the annealing time increases from 10 to 30 min, the transmittance at λ = 550 nm increases slightly from 10% to 20%. However, when the annealing time is 60 min, the transmittance at λ = 550 nm increases significantly from 20% to 68%. And at 90 min, the sample reached an average transmittance of 80%. This approach, however, requires extended periods of annealing time to remove organic materials and water from the precursor film, which is inconvenient in the actual processing process. In addition, the oscillation behavior of the transmittance spectra could be related to the optical interference due to the multilayered component [70].

Figure S4 shows that laser processing followed by 30 min of annealing allows the sample transmittance to reach up to 70%. As the scanning speed decreases from 45 mm/s to 15 mm/s, the transmittance at λ = 550 nm increases from 36% to 70% (Figure S4a); as the scanning spacing decreases from 0.08 mm to 0.02 mm, the transmittance at λ = 550 nm increases from 46% to 70% (Figure S4b). The transmittance of the laser-processed sample is higher than that of the general sample at the same annealing time. Assuming constant single pulse energy, repetition frequency, and scanning speed, it was observed that decreasing the scanning line distance incrementally increases sample transmittance. Additionally, when single pulse energy, repetition frequency, and line distance are fixed, reducing the scanning speed enhances the power density per unit area, promoting greater volatilization of organic content. Therefore, for a given annealing duration, smaller scanning line distances and slower scanning speeds yield higher transmittance. Among the parameters tested, a scanning speed of 15 mm/s and a scanning spacing of 0.02 mm were found to be optimal. However, if the scanning spacing is too small, it can damage the surface of the precursor film, as illustrated in Figure S6.

**Figure 7:**
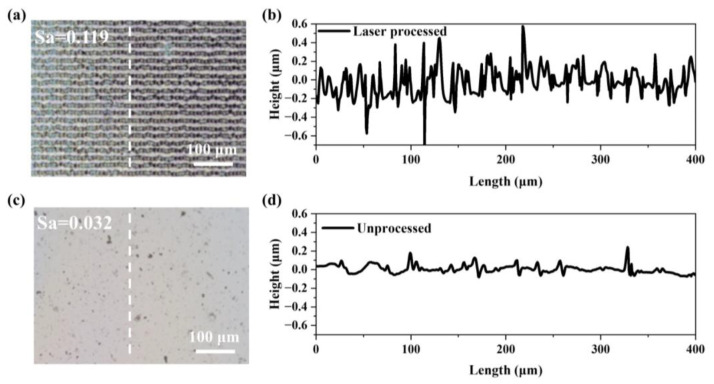
Optical images and line scan for (**a**,**b**) laser-processed surface and (**c**,**d**) unprocessed surface.

As illustrated in Figure 6b,c, under laser processing parameters for a single pulse energy of 8 μJ, a repetition frequency of 2 kHz, and varying intervals and speeds, the sample annealed for 40 min achieved a maximum transmittance of 80%. This represents a significant reduction (56%) in required annealing time—from 90 min to 40 min—when compared to unprocessed samples, achieved by optimizing the laser processing parameters. This is due to the laser-processed samples having a greater relative surface area compared to unprocessed samples, a difference underscored by their surface roughness, as depicted in Figure 7. The surface of the laser-processed sample is distributed with a periodic groove structure with a width of about 8 μm and a depth of about 0.45 μm (Figure 7a), while the surface of the laser-unprocessed sample is very smooth and flat (Figure 7c). We further measured the surface roughness (Sa) of the two samples. As shown in Figure 7b,d, the Sa of the laser-processed samples is 0.119, whereas it is only 0.032 for unprocessed samples. During heat treatment, this increased relative surface area facilitates the decomposition and volatilization of organic materials. This manifests in two key ways: firstly, the irregular micro-nanostructures on the film surface promote more uniform heating of the layer (Figure S5). The primary processing region featured particles ranging from 10–30 nm in size, whereas the secondary region exhibited strip-shaped structures with widths from 10–100 nm; secondly, compared to completely smooth surfaces, the introduction of irregular surface structures provides more sites for the volatilization of organics. Furthermore, as illustrated in Figure 6d, the sample subjected to laser processing exhibited a crystalline structure consistent with the heat treatment protocol, despite undergoing only 40 min of annealing time.

**Figure 8:**
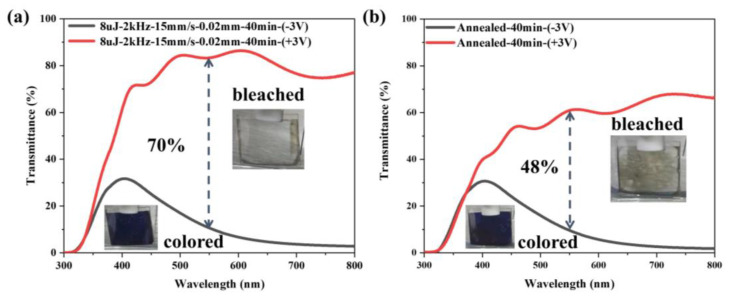
Electrochromic transmittance modulation of WO_3_ film after annealing at 500 °C for 40 min. (**a**) laser-processed sample and (**b**) general sample. In sets are the photo images of the colored state and the bleached state of samples under applied voltages for −3 V and +3 V, respectively.

Optical modulation is an important factor in evaluating the electrochromic performance. Figure 8 shows the transmission spectra of the samples in the colored (*T_c_*) and bleached (*T_b_*) states at the wavelength of 300–800 nm. The transmittance modulation *ΔT* can be calculated by the following formula:

$$\Delta T=T_{b}-T_{c}$$

For further comparison of EC performance, both the general and the laser-processed samples, subjected to a 40-min annealing time at 500 °C, were used in EC testing. As can be seen in Figure 8, when voltages of +3 V and −3 V are employed as the coloring and bleaching voltages, respectively, the laser-processed sample at 550 nm has a bleached state’s transmittance *T_b_* of approximately 80%, a colored transmittance *T_c_* of about 10%, and a transmittance modulation *ΔT* of 70%. Due to the partial volatilization of organic matter, the bleached transmittance *T_b_* of the general sample at 550 nm is only 58%, while the colored transmittance *T_c_* is basically the same as that of the laser-processed sample, and the transmittance modulation *ΔT* is approximately 48%. The results showed that the laser-processed sample exhibited a significantly higher transmittance modulation than the general sample, with a difference of approximately 22% at 550 nm. This enhancement demonstrates the efficacy of laser processing in improving the electrochromic properties of WO_3_ films with the same annealing time. In addition, the illustration in Figure 8 depicts the optical image of the colored, and bleached states. The bleached state image of the laser-processed sample demonstrates that the transmittance of the central laser-processing area is significantly higher than that of the surrounding unprocessed part, which is consistent with the experimental results.

To further demonstrate the enhanced performance of our device, we conducted a comparative test to evaluate the coloring speed between laser-processed and general WO_3_-based EC devices. The results, presented in Figure S7, indicate that the laser-processed device exhibits significantly faster coloring compared to the general device. Specifically, the laser-processed sample responds more quickly under the same applied voltage, as illustrated in Figure S7. This improved response time is attributed to the increased relative surface area of the film following laser processing, which facilitates more efficient ion insertion and extraction. The laser processing creates a more porous structure, enhancing the film’s electrochemical activity and resulting in faster and more uniform color changes. These findings suggest that laser processing not only accelerates the coloring speed but also improves the overall performance and efficiency of WO_3_-based EC devices.

## 4. Conclusions

The incorporation of femtosecond (fs) laser processing into the sol-gel fabrication of WO_3_ films represents a significant advancement in the production of electrochromic devices. This innovative method enables precise and customizable patterning of WO_3_ films, substantially enhancing their electrochromic properties. Key findings from this study include a 56% reduction in required annealing time and a 22% improvement in transmittance modulation at 550 nm, achieved by optimizing laser processing parameters. Additionally, the method improved surface roughness from Sa = 0.032 to Sa = 0.119, facilitating increased volatilization of organic material during heat treatment. These advancements underscore the potential of fs laser processing combined with the sol-gel method to significantly improve the efficiency and versatility of electrochromic devices. The enhanced surface characteristics and reduced processing time broaden the applicability of these devices in energy-efficient technologies, such as smart windows, displays, and energy storage solutions. This approach promises to pave the way for more efficient, responsive, and customizable electrochromic materials, contributing to the development of innovative energy conservation strategies.

## Data Availability

The original contributions presented in the study are included in the article, further inquiries can be directed to the corresponding author.

## Supplementary Materials

The following supporting information can be downloaded at: https://www.mdpi.com/article/10.3390/mi15060785/s1, Figure S1: WO_3_ film thickness prepared from precursor solutions with different PVP contents:(a) 0.5 g (b) 0.6 g (c) 0.8 g; Figure S2: (a) General sample and (b) solidified sample after heat treatment at 300 °C for 5 min (c) sample after heat treatment at 500 °C for 2 h (d) laser processed sample after heat treatment at 500 °C for 40 min (8 μJ-2 kHz-0.02 mm-15 mm/s); Figure S3: Different patterns obtained by processing precursor films: (a) H (b) GPL; Figure S4: Effect of different laser processing parameters and heat treatment time on sample transmittance (a,b) heat treatment at 500 °C for 30 min; Figure S5: Optical images of precursor film surface after laser processed with a line spacing of 0.01 mm; Figure S6: lower magnification of laser processed area; High magnification of the orange area; High magnification of the blue area. Figure S7: The optical images of coloring performance of (a) laser-processed (8 μJ-2 kHz-0.02 mm-15 mm/s) and (b) general WO_3_-based EC device after applying an applied (−3 V) voltage.
