# Supplementary material for: Rapid Fabrication of Tungsten Oxide-Based Electrochromic Devices through Femtosecond Laser Processing
Source: Micromachines (Basel). 2024 Jun 14;15(6):785. doi: 10.3390/mi15060785 (PMC11205389; doi:10.3390/mi15060785)
Supplement: Supplementary file 1 [file micromachines-15-00785-s001.zip › micromachines-3025578-supplementary.pdf]

Supporting Information

Rapid Fabrication of Tungsten Oxide-Based Electrochromic Devices through Femtosecond Laser Processing

Liqun Wang[1, 2], Zihao Zhai[1], Longnan Li[1, *]

[1] *GPL Photonics Laboratory, State Key Laboratory of Luminescence Science and Technology, Changchun Institute of Optics, Fine Mechanics and Physics, Chinese Academy of Sciences, 130033, Changchun, China*

[2] *Center of Materials Science and Optoelectronics Engineering, University of Chinese Academy of Sciences, Beijing, 100049, China*

*Corresponding author: Longnan Li

E-mail address: longnanli@ciomp.ac.cn

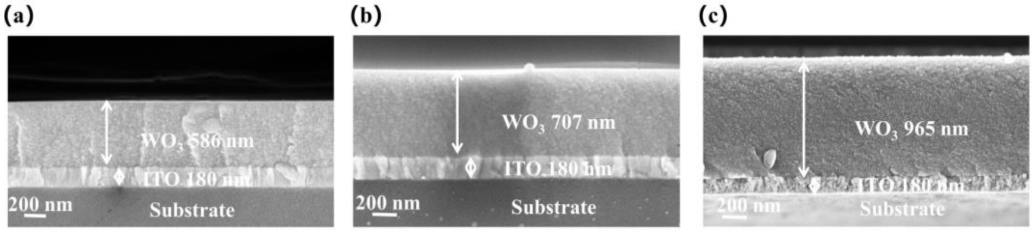

Figure S1. WO_3 film thickness prepared from precursor solutions with different PVP contents:(a) 0.5 g (b) 0.6 g (c) 0.8 g.

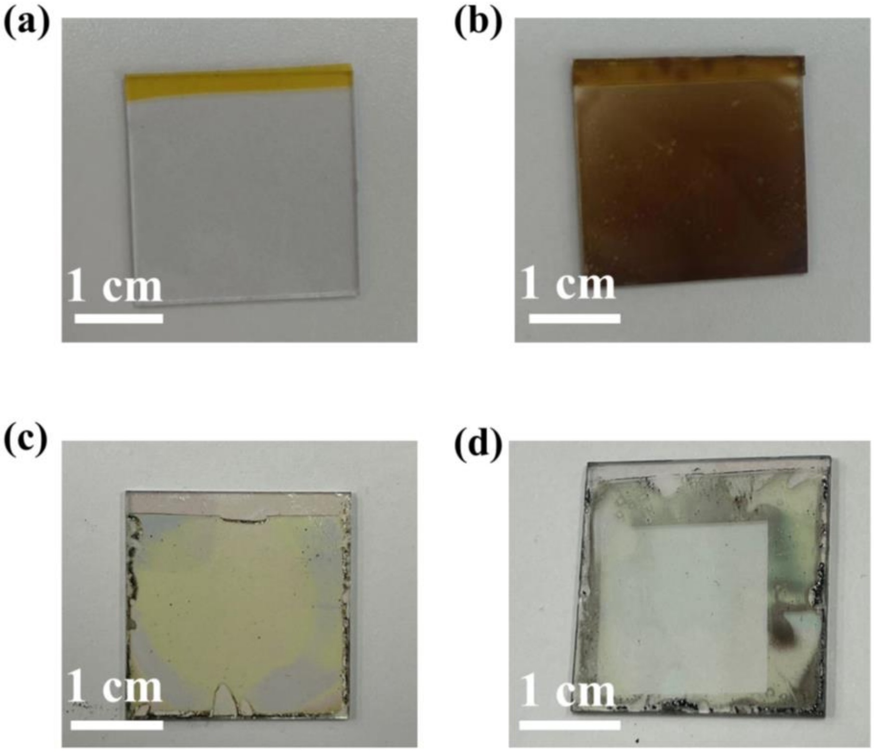

Figure S2. (a) General sample and (b) solidified sample after heat treatment at 300 ° C for 5 minutes (c) sample after heat treatment at 500 ° C for 2 hours (d) laser processed sample after heat treatment at 500 ° C for 40 minutes (8uJ-2kHz-0.02mm-15mm/s)

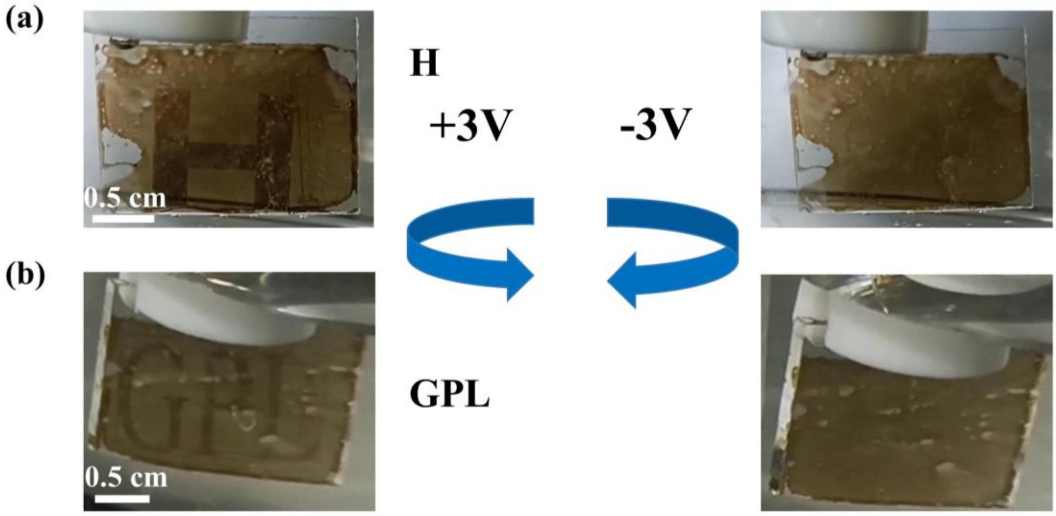

Figure S3. Different patterns obtained by processing precursor films:(a) H(b) GPL.

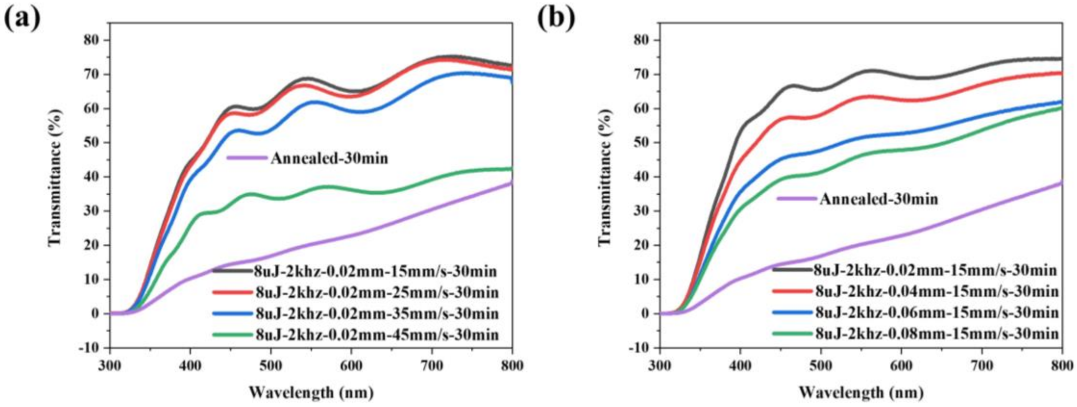

Figure S4. Effect of different laser processing parameters and heat treatment time on sample transmittance (a-b) heat treatment at 500 °C for 30 min

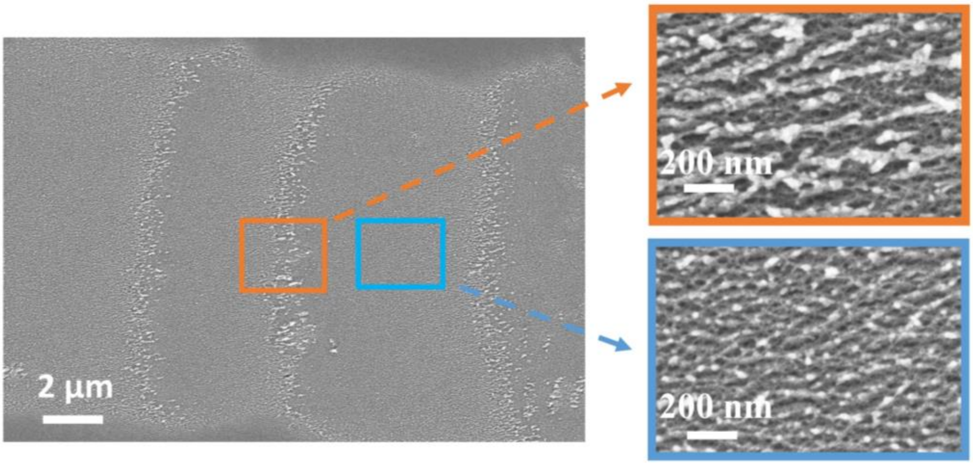

Figure S5. lower magnification of laser processed area; High magnification of the orange area; High magnification of the blue area.

8uJ-2kHz-15mm/s-0.01mm

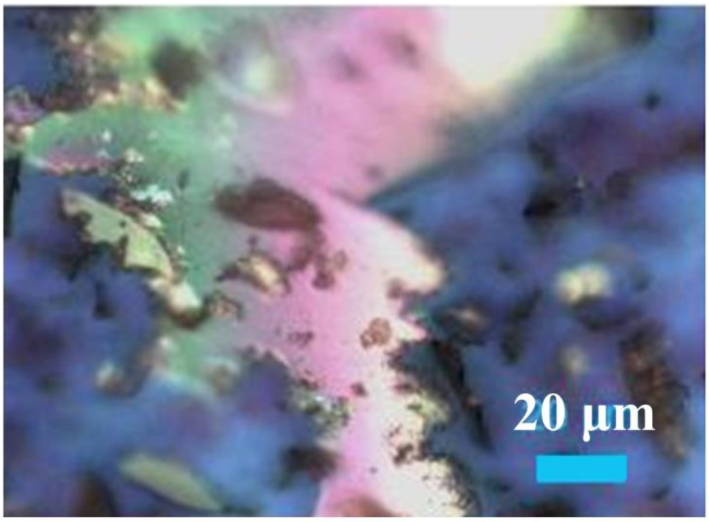

Figure S6. Optical images of precursor film surface after laser processed with a line spacing of 0.01 mm.

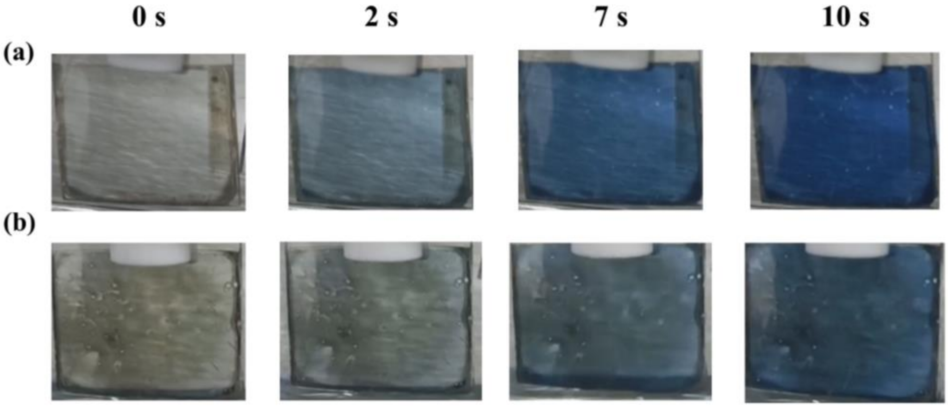

Figure S7. The optical images of coloring performance of (a) laser-processed (8 μJ-2 kHz-0.02 mm-15 mm/s) and (b) general WO3-based EC device after applying an applied (-3 V) voltage.
